# Impact of mooring activities on carbon stocks in seagrass meadows

**DOI:** 10.1038/srep23193

**Published:** 2016-03-16

**Authors:** O. Serrano, R. Ruhon, P. S. Lavery, G. A. Kendrick, S. Hickey, P. Masqué, A. Arias-Ortiz, A. Steven, C. M. Duarte

**Affiliations:** 1 School of Science and Centre for Marine Ecosystems Research, Edith Cowan University, 270 Joondalup Drive, Joondalup WA 6027, Australia; 2 UWA Oceans Institute, The University of Western Australia, 35 Stirling Highway, Crawley 6009, Australia; 3 Centro de Estudios Avanzados de Blanes, Consejo Superior de Investigaciones Científicas; Acceso a la Cala S. Francesc 14, 17300 Blanes, Spain; 4 School of Plant Biology, The University of Western Australia, Crawley 6009, Australia; 5 Research and Development Centre for Marine, Coast, and Small Islands, Hasanuddin University, Makassar, Indonesia 90245; 6 School of Earth and Environment, The University of Western Australia, 35 Stirling Highway, Crawley, WA 6009, Australia; 7 Institut de Ciència i Tecnologia Ambientals and Departament de Física, Universitat Autònoma de Barcelona, Bellaterra, 08193 Barcelona, Spain; 8 School of Physics, The University of Western Australia, 35 Stirling Highway, Crawley, WA 6009, Australia; 9 CSIRO, EcoSciences Precinct-Dutton Park 41 Boggo Road Dutton Park QLD 4102, Australia; 10 King Abdullah University of Science and Technology (KAUST), Red Sea Research Center (RSRC), Thuwal, 23955-6900, Saudi Arabia

## Abstract

Boating activities are one of the causes that threaten seagrass meadows and the ecosystem services they provide. Mechanical destruction of seagrass habitats may also trigger the erosion of sedimentary organic carbon (C_org_) stocks, which may contribute to increasing atmospheric CO_2_. This study presents the first estimates of loss of C_org_ stocks in seagrass meadows due to mooring activities in Rottnest Island, Western Australia. Sediment cores were sampled from seagrass meadows and from bare but previously vegetated sediments underneath moorings. The C_org_ stores have been compromised by the mooring deployment from 1930s onwards, which involved both the erosion of existing sedimentary C_org_ stores and the lack of further accumulation of C_org_. On average, undisturbed meadows had accumulated ~6.4 Kg C_org_ m^−2^ in the upper 50 cm-thick deposits at a rate of 34 g C_org_ m^−2^ yr^−1^. The comparison of C_org_ stores between meadows and mooring scars allows us to estimate a loss of 4.8 kg C_org_ m^−2^ in the 50 cm-thick deposits accumulated over ca. 200 yr as a result of mooring deployments. These results provide key data for the implementation of C_org_ storage credit offset policies to avoid the conversion of seagrass ecosystems and contribute to their preservation.

Among the multiple ecosystem services seagrass meadows provide, their capacity to sequester carbon dioxide has generated considerable interest among scientists and policy makers due to its potential role in helping mitigate climate change^1^,^2^,^3^. The organic carbon (C_org_) sequestered in seagrass ecosystems, along with that sequestered in other coastal vegetation such as mangroves and salt marshes, has been termed as ‘blue carbon’^4^. ‘Blue carbon’ strategies build on the opportunity to avoid or mitigate CO_2_ emissions through the conservation and restoration of seagrass meadows, which rank among the most threatened habitats in terms of global loss rates. Seagrass losses have been estimated at 29% of their global extent since 1880 and an average net decline in area of 7% yr^−1^ since 1990^5^.

Among other consequences, loss of seagrass meadows results in erosion of sediments and, potentially, the re-mineralization of the sedimentary C_org_ accumulated over millennia, which may then contribute to increasing atmospheric CO_2_^6^. Fourqurean *et al*.^1^ estimated that, considering the carbon accumulated within the top meter of seagrass meadows to be lost and remineralized upon loss of the seagrass, current rates of seagrass loss could generate emissions of up to 300 Tg carbon per year. Therefore, the destruction of seagrass habitats may contribute to the land-use component of anthropogenic CO_2_^4^.

However, the calculations above are not well constrained and there is considerable uncertainty as to the loss of C_org_ stores after meadow disturbances, a major of the bottlenecks hindering the application of seagrass conservation schemes as a low-cost method to mitigate climate change^3^,^7^,^8^. Marbá *et al*.^6^ reported that loss of a seagrass meadow in Oyster Harbor (SW Australia), led, within two decades from the loss, to the loss of the C_org_ stored in the preceding 60 years. However, this is, to the best of our knowledge, the only estimate thus far available on the loss of C_org_ stocks following seagrass loss. Hence, there is urgency in expanding the observational basis on the loss and fate of C_org_ stores following disturbances to seagrass meadows, a critical step for seagrass conservation efforts to be assigned carbon mitigation credits^8^.

Seagrasses losses derive largely from coastal eutrophication, but mechanical disturbance and destruction, such as dredging, anchoring, and coastal development are also responsible for significant losses worldwide^9^,^10^,^11^,^12^. Growing human populations and activities across many of the world’s coastlines are mirrored by an increase in the demand for mooring areas, which are often located in the sheltered locations inhabited by seagrass^13^. Moorings impact seagrass by the erosion caused by the mooring chain dragged around the meadows following wind direction and wave action, resulting in un-vegetated scars around the mooring^14^.

Even though the spatial impact of mechanical damage might not be as large-scale as other human-induced disturbances (i.e. eutrophication), moorings and anchor scars (or blowouts) can persist for decades in seagrass meadows. While seagrasses may regrow into small blowouts after moorings are removed, the un-vegetated scars can persist even decades after the original disturbance^14^. When the environmental conditions are unsuitable for seagrass re-colonization, such as in areas exposed to wave action, mooring scars act as focus for erosion, expanding in size to coalesce with adjacent ones to lead to un-vegetated blowouts^14^,^15^. This process results in seagrass fragmentation at the landscape scale, thus threatening the persistence of seagrass meadows^14^,^16^.

Australia, one of the nations supporting the highest seagrass area worldwide^17^ also has extensive mooring areas, such as that in Rottnest Island, 9 miles offshore from the city of Perth, Western Australia. The first moorings in Rottnest Island were installed in Thomson Bay in the 1930s, expanding to reach 893 moorings distributed within Rottnest embayments, with Thomson Bay supporting the largest number of moorings (316 in total;^14^,^18^). The seagrass meadows at Rottnest Island are heavily fragmented as a result of heavy use of permanent moorings^14^,^19^. To mitigate these impacts, new types of seagrass-friendly moorings have been installed from 1980s onwards (e.g. replacement of original swing moorings to single three chain cyclone moorings) that minimize the impact on seagrass, with the area of mooring-induced scars at Thomson Bay declining by 65% due to seagrass regrowth between 2004 and 2006^18^.

Whereas the impacts of the moorings on seagrass cover have been assessed^14^,^19^, the impacts on C_org_ sequestration and stocks has not been yet evaluated anywhere. Rottnest Island provides an ideal location to examine the effect of seagrass loss on the C_org_ sequestration function of seagrasses; it allows a comparison of C_org_ stores in meadows that have never been disturbed against patches that have been affected by moorings in otherwise identical environmental conditions. Here we test the hypothesis that seagrass loss and the sustained action of mooring chains lead to losses of C_org_ stocks from seagrass sediments relative to those in adjacent, undisturbed meadows.

## Results

**Figure 1:**
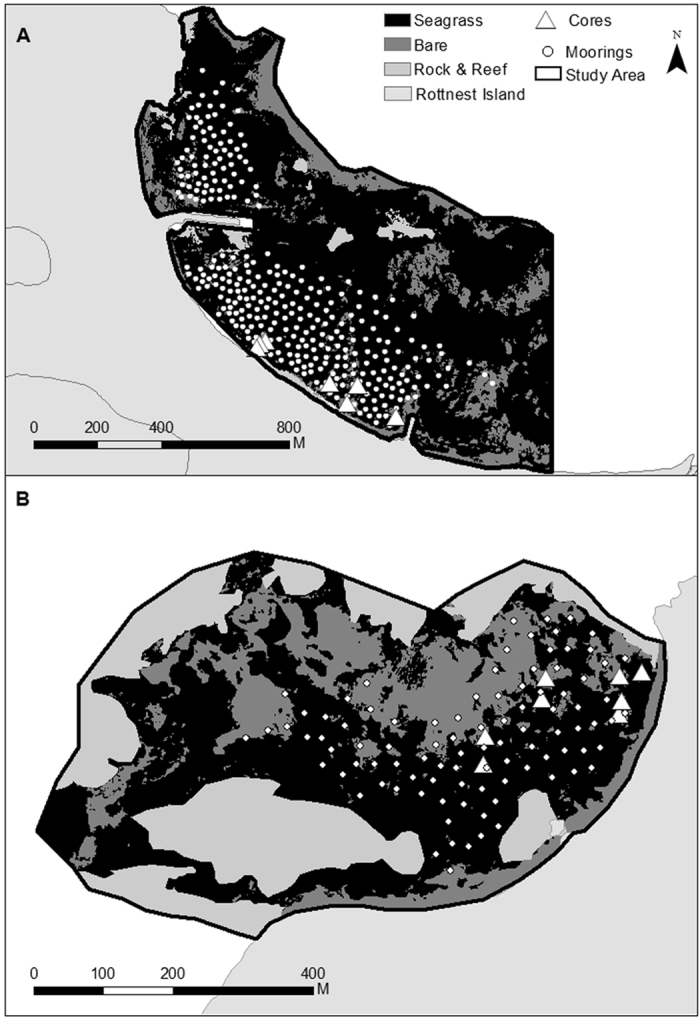
Thomson Bay (**A**) and Stark Bay (**B**) habitat maps. Three habitat area covers are used in this map: seagrass (black), bare sand (dark grey), and rock and reef (light grey). White dot points represent the mooring locations. Sediment core locations are indicated by white triangles. Habitat polygons were digitized from NearMap imagery (http://au.nearmap.com; accessed: 4^th^ July 2015; subscribed resource at University Western Australia) in ArcMap v.10.2.1. Geographic boundaries represent Australian Statistical Geography Standard (ASGS) provided by the Australian Bureau of Statistics (data freely available at http://www.abs.gov.au/AUSSTATS/abs@.nsf/Lookup/1270.0.55.001Explanatory%20Notes1July%202011?OpenDocument).

Thomson Bay, 101 ha in area, supported 80 ha of seagrass meadows, 17.2 ha of bare sand and 3.4 ha of reef. At Stark Bay, 36 ha in area, seagrass meadows covered 17.4 ha, while bare sands and reefs covered 8.5 ha and 10 ha, respectively (Fig. 1). Based on the aerial imagery obtained in 2009, a total of 316 and 95 moorings were identified at Thomson Bay and Stark Bay, respectively. The interpretation of the aerial imagery within this study showed that moorings (deployed from 1930s onwards) led to the loss of 1.3 ha and 0.9 ha of seagrass meadows at Thomson Bay and Stark Bay, respectively (Table 1). The dimensions of individual mooring scars in Stark Bay were larger and more variable (95 ± 11 m^2^) than those at Thomson Bay (41 ± 3 m^2^). Taking an average area of mooring scars of 54 ± 4 m^2^ (for both study sites) and 893 moorings in total, an estimated total area of 4.8 ha of seagrass has been lost in Rottnest Island due to moorings (Table 1).

The cores from seagrass meadows at both sites consisted of dark, brown sediments with coarse seagrass detritus (rhizomes, roots and sheaths) and shells of bivalves and gastropods embedded within the sediments. In contrast, the cores from mooring scars contained white carbonate sediments, lacking seagrass fibers in the top 15 to 25 cm, while below these depths sparse seagrass fibers were found.

**Figure 2:**
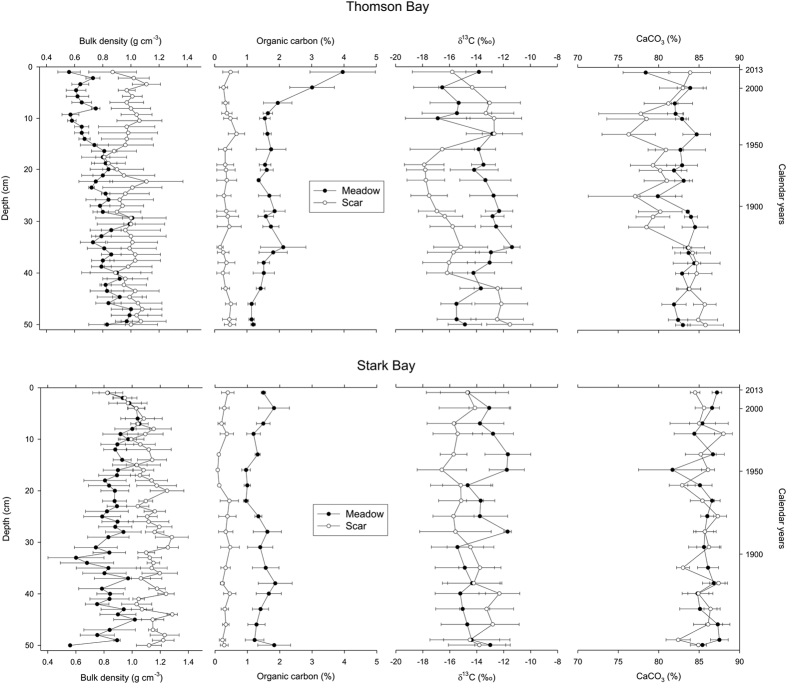
Sediment characteristics along depth in the sediment cores from seagrass meadows and mooring scars sampled at Thomson Bay and Stark Bay. Data are average ± Standard Error of Mean. Calendar years only refers to cores from seagrass meadows across both sites, based on ^210^Pb age-models.

Overall, the dry bulk density was significantly lower (*P* < 0.001) in seagrass cores (mean ± SEM; 0.82 ± 0.03 g cm^−3^) compared to the cores from the mooring scars (1.03 ± 0.03 g cm^−3^; Fig. 2; see Supplementary Table S1a and S2). The C_org_ content was five-fold higher in seagrass sediments (1.60 ± 0.06%) compared to scars (0.34 ± 0.03%; *P* < 0.001). The C_org_ content in seagrass meadows at Thomson Bay decreased from 4% in the top 2 cm to 2% at 10 cm depth to 1–2% between 10 and 50 cm (Fig. 2). The C_org_ content in seagrass meadows at Stark Bay was constant with depth (1.41 ± 0.1%), while the C_org_ content in the sediments in mooring scars was lower and constant (0.30 ± 0.04%) through the core in both sites (Fig. 2). The δ^13^C signatures of organic matter in the seagrass cores (−14.0 ± 0.3‰ at Thomson Bay and −13.8 ± 0.4‰ at Stark Bay) were heavier than those in the scars (−15.0 ± 0.4‰ at Thomson Bay and −14.6 ± 0.4‰ at Stark Bay; see Supplementary Table S1a and S2), but differences were not significant (*P* = 0.064; Table S2). No clear pattern in δ^13^C with depth was observed (Fig. 2).

**Figure 3:**
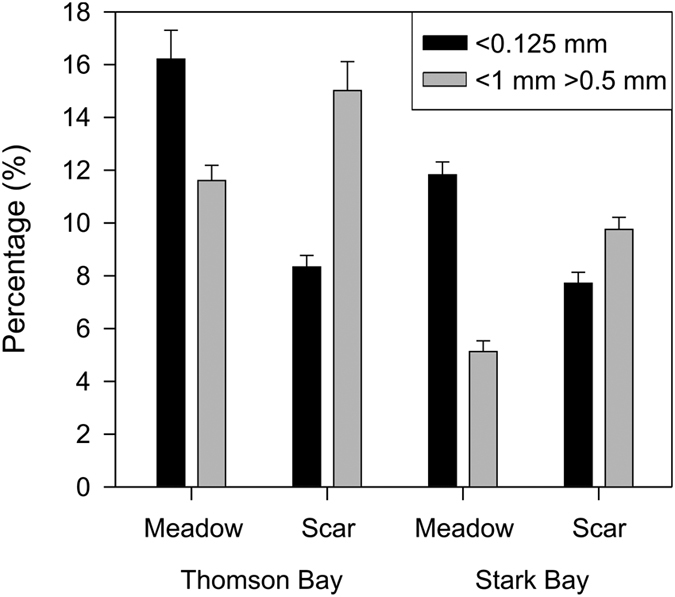
Sediment grain-size fractions, expressed as a percentage of the total sediment dry weigh <1 mm in diameter, in the sediment cores from seagrass and mooring-induced scars at Thomson Bay and Stark Bay. Sediments were classified as coarse sand (<1 mm and >0.5 mm) and very fine sand plus silt and clay (<0.125 mm).

Sediments were carbonate-rich (82–86%), and the CaCO_3_ content in seagrass cores (84%) was slightly but significantly higher than in scars (83%; *P* < 0.001; Fig. 2; see Supplementary Table S1a and S2). All the sediments were dominated by fine and medium sands (39% and 38% in seagrass meadows, and 36% and 43% in scars, respectively) but those from seagrass meadows had a significantly higher percentage of very fine sand and mud (14 ± 1%) compared to the scars (8 ± 0.4%; *P* < 0.001; Fig. 3; see Supplementary Table S2). In both sites, medium and coarse sands were significantly more abundant in the mooring scars compared to the seagrass beds (*P* < 0.001; Fig. 3), while fine sands were more abundant in seagrass meadows (*P* < 0.01; see Supplementary Table S2).

**Figure 4:**
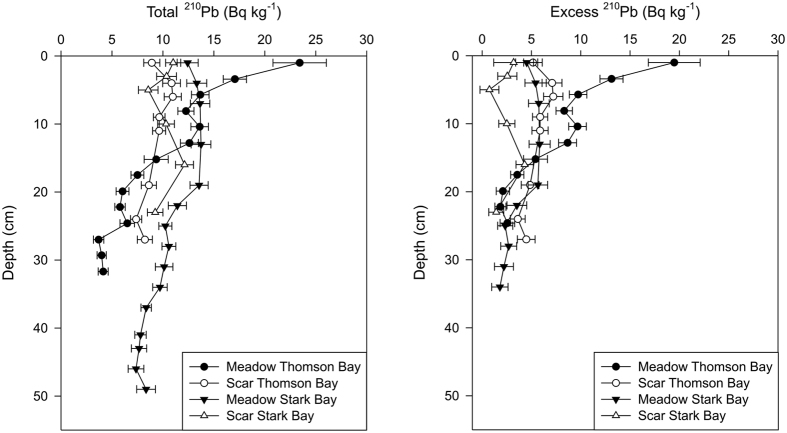
Concentration profiles of total and excess ^210^Pb in sediments of the seagrass meadows and induced mooring scars at Thomson Bay and Stark Bay.

No significant effects of sediment depth were observed for any of the variables studied (see Supplementary Table S2). Significant two-way interactions were observed for density, C_org_ and CaCO_3_ inventories, very fine sand and mud, and medium sands. Excess ^210^Pb horizons in seagrass meadows in Thomson Bay and Stark Bay were reached at 26 cm and 35 cm depth, yielding average sedimentation rates of 0.25 ± 0.01 and 0.27 ± 0.08 cm yr^−1^, respectively (Fig. 4). However, the seagrass core at the Stark Bay site showed evidence of mixing in the upper 15 cm, and thus the estimated sedimentation rate must be considered an upper limit. Sediment cores collected from mooring scars in both sites showed constant concentrations of ^210^Pb of about 10 Bq·kg^−1^, not making it possible to estimate reliable sedimentation rates. Supported ^210^Pb concentrations in the scar areas were of 3.8 ± 0.5 and 10 ± 1 Bq·kg^−1^ in the Thomson Bay and Stark Bay sites, respectively, in agreement with those from seagrass meadows. The negligible amount of excess ^210^Pb in Stark Bay suggests that significant erosion of the sediments had occurred in scar areas in the past, while the uniform excess ^210^Pb in Thomson Bay down to 30 cm depth suggests deep mixing and intense redistribution of the sediment. In contrast, the inventory of excess ^210^Pb in the scar site at Stark Bay was negligible, since concentrations of ^210^Pb were not statistically different from supported ^210^Pb concentrations, indicating substantial erosion at this site. The excess ^210^Pb inventories in sediments from seagrass meadows were very similar, 1201 ± 55 Bq·m^−2^ at Thomson Bay and 1250 ± 103 Bq·m^−2^ at Stark Bay, corresponding to an annual atmospheric flux of 38 ± 2 Bq·m^−2^·yr^−1^. This flux is in good agreement with reported atmospheric fluxes of ^210^Pb in nearby Perth (43 Bq·m^−2^·yr^−1^; 20). This agreement support the hypothesis that sediments under seagrass meadows contained all deposited excess ^210^Pb for the last 100–150 years.

**Figure 5:**
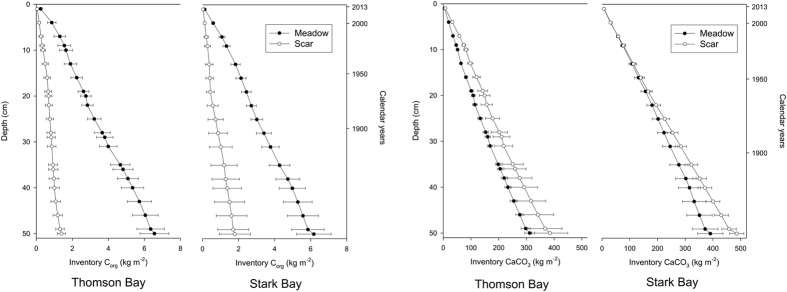
Mean ± SEM inventory of organic carbon (C_org_) and calcium carbonate (CaCO_3_) in sediments from seagrass meadows and mooring-induced scars at Thomson Bay and Stark Bay. Calendar years only refers to cores from seagrass meadows across both sites, based on ^210^Pb age-models.

The C_org_ inventories (normalized to 50 cm-thick deposits) in the mooring scars (1.4 ± 0.2 at Thomson Bay and 1.8 ± 0.9 kg C_org_ m^−2^ at Stark Bay) were four- to five-fold lower than in adjacent seagrass meadows (6.6 ± 0.8 at Thomson Bay and 6.2 ± 0.9 kg C_org_ m^−2^ at Stark Bay; Fig. 5; see Supplementary Table S1b and S2). Based on the estimated sedimentation rates, the seagrass meadows at Thomson Bay and Stark Bay accumulated 34 ± 3 and 34 ± 10 g C_org_ m^−2^ yr^−1^, respectively (see Supplementary Table S1b). The seagrass meadows at both study sites stored somewhat lower amounts of CaCO_3_ in their sediments (312 ± 18 kg CaCO_3_ m^−2^ and 391 ± 46 kg CaCO_3_ m^−2^, respectively) compared to mooring scars (384 ± 65 kg CaCO_3_ m^−2^ and 486 ± 26 g CaCO_3_ m^−2^; see Supplementary Table S1b and S2). Seagrass meadows at both study sites accumulated, on average, 1.6 ± 0.1 kg CaCO_3_ m^−2^ yr^−1^ and 2.0 ± 0.6 kg CaCO_3_ m^−2^ yr^−1^, respectively (see Supplementary Table S1b).

## Discussion

Mechanical destruction of seagrass meadows by moorings at Rottnest Island from the 1930s onwards has led to the loss of their C_org_ sequestration capacity by precluding further accumulation of C_org_, and through the erosion of the centenary C_org_ stores underneath seagrass meadows. The deployment of moorings since the 1930s had led to an average loss of at least 4.8 kg C_org_ m^−2^ that had accumulated in 50 cm-thick deposits over the last ca. 200 years. Because the undisturbed meadows contained up to 2-fold higher amounts of fine sediments (<0.125 mm) compared to mooring scars, it appears that the loss of C_org_ resulted from both direct scouring of sediments and re-suspension and subsequent loss of fine-grained sediments. The presence of fine-grained sediments in the substrate contributes to the accumulation and preservation of C_org_, due to their higher capacity to retain organic matter compared to coarse sediments^21^, and because fine-grained sediments contribute to the creation of anoxic conditions in the sediment^22^ and, ultimately, to the higher preservation of C_org_ through reduced remineralization rates^23^,^24^. The seagrass canopy has the ability to trap fine sediments suspended in the water column, reducing wave energy and resuspension^25^. Furthermore, the presence of seagrass rhizomes and roots stabilizes sediments and reduces resuspension^26^. Mooring scars with no seagrass fibers in the sediment, living or dead, tend to be unstable, leading to further erosion of the scar edges while creating deeper scouring areas^15^,^27^ and ultimately, contribute to greater loss of sedimentary C_org_ stores.

The enriched δ^13^C signatures of organic matter present in the seagrass meadows indicate a smaller contribution of seagrass-derived organic matter to the sediments in mooring scars. The uniform concentration of excess ^210^Pb and its inventory indicate that sediments had not been accumulated at the scar site in Thomson Bay, but likely eroded and thoroughly mixed. Given that the accumulated stocks of C_org_ at mooring scars in Thomson Bay were significantly lower than in seagrass meadows, it is likely that intense sediment reworking through disturbance probably led to the net loss of C_org_ stocks. On the other hand, the depletion of excess ^210^Pb in the scar site at Stark Bay suggests that erosion of sediments, and loss of the associated C_org_, were significant after seagrass meadows had been lost due to mooring activities. Therefore, the lack of sediment accumulation as a consequence of mooring deployment and scouring of the living seagrass biomass may also lead the loss of their C_org_ sequestration capacity by precluding further accumulation of sediments and C_org_, estimated at 34 g C_org_ m^−2^ yr^−1^ (based on ^210^Pb age-models). Over an average 50-year period of mooring presence at Rottnest Island^18^, the loss of C_org_ accumulation following disturbance has been estimated as 1.7 kg C_org_ m^−2^. Note that the C_org_ stores in the living biomass were also loss due to mooring activities, but were not accounted for in this study.

Examination of the vertical profile of C_org_ relative to the estimated sediment date shows that loss of seagrass cover by the mooring activity led to erosion and subsequent loss of the C_org_ stored over the past ca. 200 years. This is a far more severe erosive loss than that reported by Marbà *et al*.^6^ in Oyster Harbor (SW Australia) following eutrophication, where 60 years of C_org_ accumulation was lost 25 yr after seagrass loss occurred. Hence, the results presented here show that the movement of mooring chains induce sediment erosion and a considerable loss of C_org_ stores, which are then exposed to oxic conditions that in turn may lead to remineralization of organic matter^28^, returning sedimentary C_org_ stocks to the ocean and atmosphere in the form of CO_2_.

The analysis of aerial imagery from 2009 showed that moorings produced scars averaging 41 m^2^ at Thomson Bay and much larger, 95 m^2^, at Stark Bay. The differences in the sizes of scars at the two sites may reflect a number of processes. The length of chain on the mooring and scouring from propeller wash from large boats may lead to initially different sized scars. Subsequent environmental processes (i.e. regrowth or erosion) will then act on the scar and can vary depending on the geomorphology and the hydrodynamic energy at each site^13^,^29^,^30^. A third factor is the period of time the scar has been present (or deployment time of the mooring), since this will allow greater opportunity for erosional processes to act. Thomson Bay is more sheltered than Stark Bay^14^, where the edges of mooring-induced scars would erode further due to wave action resulting in coalescence between adjacent mooring scars. It is possible to determine from the 1941 and 2003 aerial images that the mooring scars sampled at Thomson Bay have always been present since 1941. However, two out of the four mooring scars sampled at Stark Bay were seagrass back in 1941, but scars in 2003. The resolution of the 1941 imagery is very low (not shown), and the lack of information about the history of mooring deployments at Rottnest Island precludes further interpretations on the effects of time and type of mooring on C_org_ loss.

According to Walker *et al*.^19^, scars originated from mooring activities in the 1980s at Thomson Bay and Stark Bay averaged 71 and 17 m^2^ in diameter and caused a loss of 2.4 ha and 0.18 ha, respectively (Table 1). Though the size of mooring-induced scars observed here contrasted with those reported by Walker *et al*.^19^, the apparent decrease in the average scarring at Thomson Bay could indicate recovery at this site. According to Rottnest Island Authority^18^, the area of mooring-induced scars at Thomson Bay experienced a 65% decrease (i.e. seagrass growth) during the period of 2004 to 2006. Although the methods and imagery used to estimate the seagrass area loss were not the same, our results seem to indicate that the efforts undertaken by Rottnest Island Authority to reduce the impacts of moorings by replacing some moorings for more environmentally-friendly ones^18^ is contributing to reduce the area of mooring scars at Thomson Bay. On the other hand, the scar size measured in this study at Stark Bay is 5 to 6-fold higher than those measured by Walker *et al*.^19^ (Table 1), pointing at the coalescence and blowout expansion of initial patches originated by moorings^13^,^15^. In particular, this phenomenon is more evident in the northern, deeper parts of Stark Bay, which are further offshore and more exposed to hydrodynamic energy.

Although our sample size was small (i.e. 4 cores per site and treatment), the results presented demonstrate that loss of seagrasses by mooring activities in Rottnest Island led to significant loss of 75%, on average, of the sedimentary C_org_ stocks accumulated in the top 50 cm of sediment, in addition to loss of the seagrass C_org_ sequestration capacity. These losses were associated with erosion of fine sediments leading to the loss of much of the C_org_ stock accumulated over the past 200 yr, thereby involving the loss of C_org_ accumulated about 160 years prior to the loss of seagrass. Although the fate of C_org_ lost after disturbances remain unknown, the C_org_ stores are eroded and at risk to be re-mineralized in the water column or surface sediments onto which they may be re-deposited. Our results add to the report by Marbà *et al*.^6^, for seagrass loss following eutrophication, to demonstrate that seagrass loss leads to the loss of much of the C_org_ stored over decades and even centuries.

Considering the impact of mooring activities on coastal areas and its capacity to disturbing the sequestration and preservation of C_org_ in seagrass meadows, among other ecosystem services that they provide, there is an urgent need for better management focused on mooring areas. Since seagrass rehabilitation is costly and relatively unsuccessful in southern Australia^31^, the use of environmentally-friendly moorings might be a plausible solution to the problem.

## Material and Methods

### Study site, sampling and laboratory procedures

**Figure 6:**
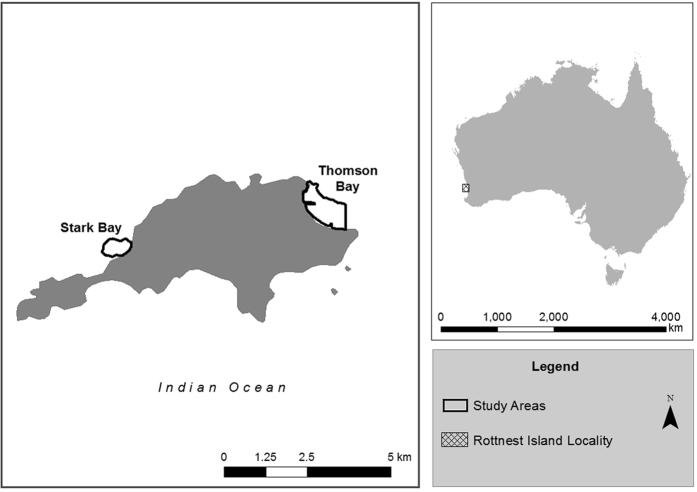
Rottnest Island is located 18 km off the coast of Perth, Western Australia. The black outline depicts the study regions within Thomson Bay and Stark Bay. Geographic boundaries represent Australian Statistical Geography Standard (ASGS) provided by the Australian Bureau of Statistics (data freely available at http://www.abs.gov.au/AUSSTATS/abs@.nsf/Lookup/1270.0.55.001Explanatory%20Notes1July%202011?OpenDocument). Map generated in ArcMap v10.2.1.

Sampling was conducted at Thomson Bay (31°59′-32°00′S, 115°32′E) and Stark Bay (32°00′S, 115°29′E), Rottnest Island (Western Australia; Fig. 6). Rottnest Island is a marine reserve, surrounded by limestone reefs and extensive sub-tidal sand and seagrass habitats^18^. Rottnest Island is a popular tourism destination, with approximately 350,000 visitors per year in the 1990s, of which 70,000 arrived on private vessels^32^. By 2009, the number of visitors increased to approximately half a million, with 150,000 arriving by private vessel^29^. The Australian Army set up moorings for explosives barges during World War II. Some of these original moorings were converted to recreational moorings in the 1950s and 1960s. An increase in the number of moorings occurred over the 1970s and early 1980s, resulting in the establishment of a moratorium on moorings. From the 1980s onwards, new environmentally friendly moorings were installed. Damage produced by mooring chains dragged over the meadows denuded these areas of seagrass, resulting in sandy patches within the seagrass beds^19^, which were first reported from an aerial photograph in 1941^14^.

In order to assess the impacts of moorings on C_org_ stocks, a total of 16 sediment cores were sampled: four sediment cores in mooring scars and four cores in adjacent and undisturbed mixed meadows of *Posidonia sinuosa* and *Amphibolis griffithii* at each study site (Thompson Bay and Stark Bay; Fig. 1). Sediments were sampled using PVC cores (80 cm long, 6 cm in diameter) that were gently hammered into the sediment at 2 to 4 m water depth.

PVC cores were cut lengthwise, and sediments inside the cores were cut into 1 cm-thick slices. Each slice was weighed before and after oven drying at 60 °C until constant weight (dry weight; DW). Then, every second slice was divided into two subsamples by quartering (i.e. 18–22 slices per core). One subsample was ground and analyzed for organic carbon (C_org_) and stable isotope composition (δ^13^C), and calcium carbonate (CaCO_3_) content. The other subsample was used for sediment grain-size analyses.

For C_org_ and δ^13^C analyses, 1 g of ground sample was acidified with HCl 4% until bubbling stopped to remove inorganic carbon, centrifuged (3400 rpm for 5 minutes), and the supernatant with acid residues removed carefully by pipette, avoiding resuspension of the sediment. Then, the sample was washed with Milli-Q water, centrifuged, and the supernatant removed. The residual samples were re-dried and encapsulated for analysis using a Micro Cube elemental analyzer (Elementar Analysensysteme GmbH, Hanau, Germany) interfaced to a PDZ Europa 20-20 isotope ratio mass spectrometer (Sercon Ltd., Cheshire, UK) at the University of California Davis. Content of C_org_ was calculated for bulk (pre-acidified) samples.

The CaCO_3_ content was analyzed with a Calcimeter (Pressure Gauge Model 432 (Fann®); ASTM D 4373-84 Standard) by adding 10% HCl to the sample in a sealed reaction cell. The pressure build up due to the CO_2_ was measured with a bourdon tube pressure gauge that was pre-calibrated with reagent grade calcium carbonate.

For sediment grain-size analysis, a Mastersizer 2000 laser-diffraction particle analyzer was used following digestion of the samples with hydrogen peroxide to remove organic matter. Sediments were classified as coarse sand (<1 mm and >0.500 mm) medium sand (<0.500 mm and >0.250 mm), fine sand (<0.250 mm and >0.125 mm), and very fine sand plus mud (<0.125 mm), according to a scale adapted from Brown and McLachland^33^.

Two sediment cores per site (i.e. one core from a mooring scar and one from adjacent meadows) were analyzed for ^210^Pb concentration to determine recent (ca. 100 years) sedimentation rates. ^210^Pb was determined through the analysis of ^210^Po by alpha spectrometry after addition of ^209^Po as an internal tracer and digestion in acid media using an analytical microwave^34^. The concentrations of excess ^210^Pb used to obtain the age models were determined as the difference between total ^210^Pb and ^226^Ra (supported ^210^Pb). Concentrations of ^226^Ra were determined for selected samples along each core by low-background liquid scintillation counting method (Wallac 1220 Quantulus) adapted from Masqué *et al*.^35^. These concentrations were found to be in agreement with the concentrations of total ^210^Pb at depth below the excess ^210^Pb horizons. Mean sedimentation rates over the last 100 years were determined using the CF:CS model^36^.

### Data analyses

Buoys attached to boat moorings at Stark Bay and Thomson Bay were digitized from high-resolution aerial imagery obtained in October 2009 (NearMap, http://au.nearmap.com) using the Geographic Information System (GIS) software ESRI ArcGIS. The bays were initially divided into a 20 × 20 m grid. Three coverage types were identified within each grid cell: seagrass meadows, bare sediments and rock/reef structures, which were digitized at a 1:25 scale. The total area of seagrass meadows, bare sediments, reef structures, and mooring induced scars was then calculated for each bay (Fig. 1).

**Figure 7:**
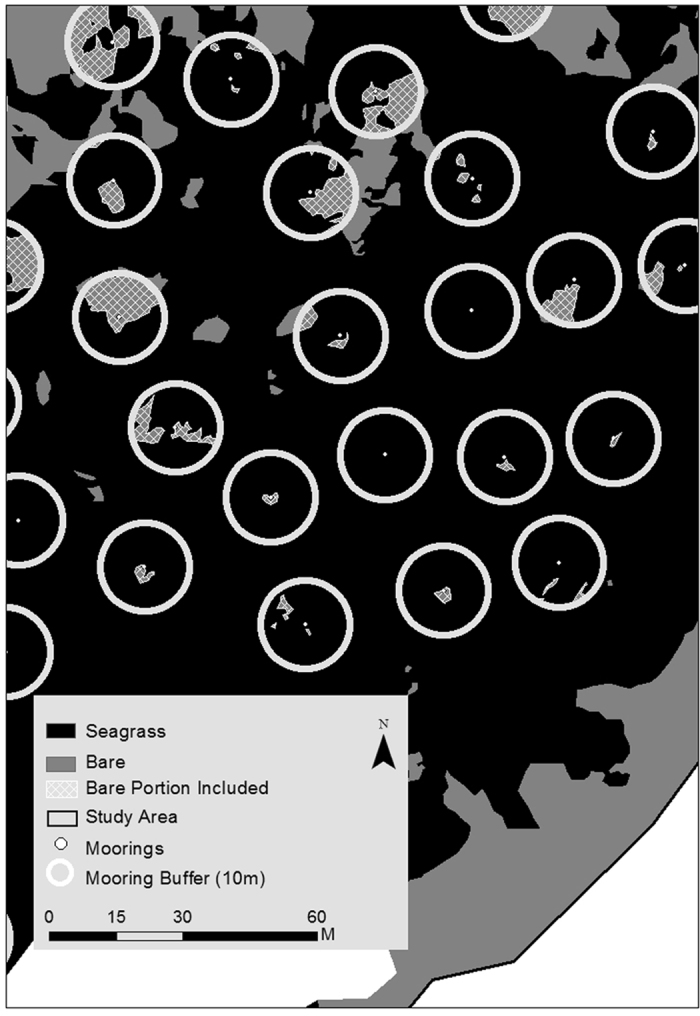
Mapping of mooring scars at Thomson Bay. Two habitat area covers are used in this map: seagrass (black) and bare sand (dark grey). White dot points represent the mooring locations. The 10 m buffer around moorings generated within ArcMap v.10.2.1 is indicated by white circles. The bare sediment portion within the 10 m of the digitized buoys used for the computations of mooring-induced scars is indicated by diagonal crosshatches. Geographic boundaries represent Australian Statistical Geography Standard (ASGS) provided by the Australian Bureau of Statistics (data freely available at http://www.abs.gov.au/AUSSTATS/abs@.nsf/Lookup/1270.0.55.001Explanatory%20Notes1July%202011?OpenDocument). Habitat polygons were digitized from NearMap imagery (http://au.nearmap.com; accessed: 4^th^ July 2015; subscribed resource at University Western Australia) in ArcMap v.10.2.1.

The area of mooring-induced scarring was calculated for each digitized buoy to a Euclidean buffer of 10 meters. A radius of 10 m was chosen based on the average drifting lengths of the buoys with respect to their anchoring point in the seafloor at our study sites, and encompasses the length of mooring chain and possible dragging into the sediment. Currents and waves displace the buoy from its anchoring point (i.e. the chain attached to the buoy is not perpendicularly aligned to the seafloor) and we estimated (from aerial imagery) that the drifting lengths within the study area digitised average 10 m radius. As such there is an assumption that bare areas within this buffer are mooring induced, and that bare areas outside this buffer are not mooring induced. The spatial analyst toolbox within ArcMap was used to calculate the 10 m intersect area and determine the portion of any ‘bare sediment’ polygon that was located within 10 m of the digitized buoys (Fig. 7).

To allow direct comparisons among treatments and sites, the standing stocks per unit area (cumulative stocks; kg DW m^−2^) were standardized to 50 cm-thick deposits (i.e. average length of the sediment cores sampled; extrapolated when necessary). The length of core barrel inserted into the sediment and the length of retrieved seagrass sediment were recorded in order to correct the core lengths for compression effects and all variables studied here are referenced to the corrected, uncompressed depths. The mooring-induced loss of C_org_ stocks was determined from the difference between the average accumulated stocks of C_org_ in seagrass meadows and scar areas. Sediment accumulation rates (g DW m^−2^ y^−1^) for the last century were estimated from the ^210^Pb age models. Accumulation rates of C_org_ were estimated by dividing the inventories in the 50 cm-thick sediments by the average sediment accretion rate derived from ^210^Pb (i.e. assuming constant sediment accretion rates through the core). For the three replicate cores, which were not ^210^Pb dated, it was assumed they had the mean accumulation rate of the dated core at the site and treatment (i.e. mooring scar and undisturbed meadow).

All analyses were performed using Generalized Linear Mixed Model procedures in SPSS v. 14.0. A Generalized Linear Mixed Model was used to take into account the potential non-independence of samples taken at different depths within the same core, since depth is a proxy for time in the cores. Given the spatial separation of cores within meadows (hundreds of meters) we considered the cores themselves to be spatially independent. All response variables (bulk density, C_org_ and CaCO_3_ inventories, δ^13^C signatures and sediment grain size fractions were square-root transformed prior to analyses and had homogenous variances. Treatment (meadows *vs* scars) and sediment depth (18 depths along 50 cm-thick sediment cores) were treated as fixed factors and study site (Thompson Bay *vs* Stark Bay) was included as a random effect in all statistical models (probably distribution: normal; link function: identity).

## Additional Information

**Data availability**: Data supporting this study are available in this link: http://ro.ecu.edu.au/datasets/26/.

**How to cite this article**: Serrano, O. *et al*. Impact of mooring activities on carbon stocks in seagrass meadows. *Sci. Rep*. **6**, 23193; doi: 10.1038/srep23193 (2016).

## Supplementary Material

Supplementary Information

## Figures and Tables

**Table 1:** Seagrass and mooring scar areas at Rottnest Island.

Site	N moorings	Total seagrass area (ha)	Average size scar (m^2^)	Total mooring scar area (ha)
Thomson Bay	344	93	70.9	2.44
	**316**	**80**	**41**	**1.3**
Stark Bay	105	16	17.4	0.18
	**95**	**17**	**95**	**0.9**
Catherine Bay	31	8.1	8.1	0.03
Rocky Bay	191	31.9	12.5	0.24
Geordie bay	92	10.5	7.8	0.07
Longreach Bay	73	10.2	8.1	0.06
Porpoise bay	33	28.6	3.9	0.01
Marjorie bay	64	24.5	16.6	0.11
Total	933	223	33.6	3.13
Total*	893*	–	**54**	**4.8**

Estimated by Walker *et al*.^19^ (based on 1980 imagery) and in this study (based on 2009 imagery, marked in bold).

^*^RIA, 2014.
