# Supplementary material for: Impact of mooring activities on carbon stocks in seagrass meadows
Source: Sci Rep. 2016 Mar 16;6:23193. doi: 10.1038/srep23193 (PMC4793266; doi:10.1038/srep23193)
Supplement: Supplementary Information [file srep23193-s1.pdf]

Supplementary information

Impact of mooring activities on carbon stocks in seagrass meadows

Serrano O., R. Ruhon, P. Lavery, G. A. Kendrick, S. Hickey, P. Masqué, A. Arias-Ortiz, A. Steven, C.M. Duarte

Supplementary Table S1. Descriptive statistics of the variables studied in the sediment cores.

a)

Site	treatment	N	Density ($g\ cm^{-3}$)		$\delta^{13}C$ (‰)		C_{org} (%)		$CaCO_3$ (%)	
			mean	SEM	Mean	SEM	Mean	SEM	Mean	SEM
Thomson Bay	meadow	88	0.78	0.02	-13.96	0.32	1.76	0.10	82.84	0.32
	scar	88	0.98	0.03	-15.00	0.42	0.38	0.04	81.57	0.64
Stark Bay	meadow	72	0.87	0.03	-13.81	0.39	1.41	0.07	85.80	0.39
	scar	72	1.09	0.02	-14.62	0.45	0.30	0.04	85.38	0.36

N, number of analysis. SEM, Standard Error of the Mean. The calcium carbonate ($CaCO_3$) and organic carbon (C_{org}) are expressed as a percentage of the total sample dry weight.

b)

Site	treatment	Inventory (Kg $C_{org}\ m^2$)		Accumulation rates (^{210}Pb) (g $C_{org}\ m^2\ yr^{-1}$)		Inventory (Kg $CaCO_3\ m^2$)		Accumulation rates (^{210}Pb) (g $CaCO_3\ m^2\ yr^{-1}$)	
		Mean	SEM	Mean	SEM	Mean	SEM	Mean	SEM
Thomson Bay	meadow	6.6	0.8	33.9	2.9	312	18	1596	99
	scar	1.4	0.2	-	-	384	65	-	-
Stark Bay	meadow	6.2	0.9	33.6	9.9	391	46	2045	594
	scar	1.8	0.9	-	-	486	26	-	-

$CaCO_3$ and C_{org} inventories and accumulation rates calculated for 50 cm-thick sediment deposits.

Supplementary Table S2. Results from the Generalized Linear Mixed Models. Sediment density, organic carbon (C_{org}) and calcium carbonate ($CaCO_3$) inventories, stable carbon isotope signatures ($\delta^{13}C$) of sedimentary organic matter, and sediment grain size fractions in response to treatment (meadow vs scar) and sediment depth (cm) (fixed effects). Study site (Thompson Bay and Stark Bay) was included as a random factor in all analyses. The degrees of freedom (d.f.) for each term in the mixed model analysis are indicated.

Variable	Factor	*F*	d.f.	*P*-value
**Density (g cm^{-3})**	Treatment	47.663	1	**<0.001**
	Depth	0.698	17	0.805
	Treat x Depth	3.307	17	**<0.001**
**C_{org} inventory**	Treatment	37.075	1	**<0.001**
	Depth	0.881	17	0.597
	Treat x Depth	21.669	17	**<0.001**
**$CaCO_3$ inventory**	Treatment	40.524	1	**<0.001**
	Depth	0.717	17	0.785
	Treat x Depth	2.928	17	**<0.001**
**$\delta^{13}C$ (‰)**	Treatment	3.463	1	*0.064*
	Depth	0.406	17	0.984
	Treat x Depth	0.576	17	0.916
**<0.125 mm (%)**	Treatment	92.556	1	**<0.001**
	Depth	0.502	17	0.951
	Treat x Depth	5.616	17	**<0.001**
**>0.125<0.25 mm (%)**	Treatment	9.693	1	**0.002**
	Depth	0.278	17	0.998
	Treat x Depth	0.801	17	0.699
**>0.25<0.5 mm (%)**	Treatment	34.844	1	**<0.001**
	Depth	0.436	17	0.976
	Treat x Depth	2.347	17	**0.002**
**>0.5<1 mm (%)**	Treatment	31.367	1	**<0.001**
	Depth	0.502	17	0.951
	Treat x Depth	2.217	17	**0.004**

Note: The effects of the random factor were nonsignificant in all cases
